# Heart Rate Detection Using Microsoft Kinect: Validation and Comparison to Wearable Devices

**DOI:** 10.3390/s17081776

**Published:** 2017-08-02

**Authors:** Ennio Gambi, Angela Agostinelli, Alberto Belli, Laura Burattini, Enea Cippitelli, Sandro Fioretti, Paola Pierleoni, Manola Ricciuti, Agnese Sbrollini, Susanna Spinsante

**Affiliations:** Dipartimento di Ingegneria dell’Informazione, Università Politecnica delle Marche via Brecce Bianche 12, Ancona 60131, Italy; a.agostinelli@univpm.it (A.A.); a.belli@univpm.it (A.B.); l.burattini@univpm.it (L.B.); e.cippitelli@univpm.it (E.C.); s.fioretti@univpm.it (S.F.); p.pierleoni@univpm.it (P.P.); m.ricciuti@staff.univpm.it (M.R.); a.sbrollini@pm.univpm.it (A.S.); s.spinsante@univpm.it (S.S.)

**Keywords:** heart rate, contactless sensing, EVM, Kinect, RGB-D sensors, photoplethysmography, videoplethysmography

## Abstract

Contactless detection is one of the new frontiers of technological innovation in the field of healthcare, enabling unobtrusive measurements of biomedical parameters. Compared to conventional methods for Heart Rate (HR) detection that employ expensive and/or uncomfortable devices, such as the Electrocardiograph (ECG) or pulse oximeter, contactless HR detection offers fast and continuous monitoring of heart activities and provides support for clinical analysis without the need for the user to wear a device. This paper presents a validation study for a contactless HR estimation method exploiting RGB (Red, Green, Blue) data from a Microsoft Kinect v2 device. This method, based on Eulerian Video Magnification (EVM), Photoplethysmography (PPG) and Videoplethysmography (VPG), can achieve performance comparable to classical approaches exploiting wearable systems, under specific test conditions. The output given by a Holter, which represents the gold-standard device used in the test for ECG extraction, is considered as the ground-truth, while a comparison with a commercial smartwatch is also included. The validation process is conducted with two modalities that differ for the availability of a priori knowledge about the subjects’ normal HR. The two test modalities provide different results. In particular, the HR estimation differs from the ground-truth by 2% when the knowledge about the subject’s lifestyle and his/her HR is considered and by 3.4% if no information about the person is taken into account.

## 1. Introduction

The measurement and monitoring of vital signs, such as Heart Rate (HR) and blood pressure, are of interest to the healthcare system [1], mainly because they can help with diagnosis and follow-up of different medical issues, including heart diseases. The HR, which is the number of heart beats in one minute, is a parameter affected by the level of activity or stress and also by the age of a subject. According to Table 1, adults at rest feature an HR between 60 and 80 beats per minute (bpm). The HR of athletes may drop to 40 bpm at rest, because the heart muscle is stronger and has greater output. Teens usually feature an HR higher than 70 bpm, while HR in infants can reach 180 bpm during crying [2]. Adults involved in physical activity can show an HR within the range of 150–200 bpm.

Standard methods for heartbeat detection rely on the use of the Electrocardiograph (ECG), which is an expensive and uncomfortable device, the use of which is typically limited to clinical premises [3,4]. Nowadays, the availability of gadgets for lifestyle logging that are easier to use is increasing. They include custom [5] or commercial devices, since the HR estimation capability is also provided by some smartwatches and smartphone applications [6]. Recently developed technologies [7,8,9,10,11,12] implement different strategies to have non-contact and non-invasive systems for the analysis of neurological and sleep disorders, stress assessment, evaluation of human activity and also HR estimation. The HR contactless detection [13,14] offers technological and scientific progress in the diagnostic and healthcare fields, but also for everyday use at home.

The adoption of a Microwave Impulse Radar (MIR) may represent a possible approach, being based on the analysis of small and almost undetectable body movements caused by a varying flow in the blood vessels [15,16]. On the other hand, HR detection through video processing is mainly based on extremely small variations of skin color, which are not perceptible to the human eye, caused by the flow of blood in the tissues. Since the oxygenated blood flow changes the amount of hemoglobin molecules and proteins, these changes also affect the light optical absorption, making it possible to identify the HR on the basis of the color changes of the skin. By illuminating the skin and measuring the amount of light transmitted or reflected, it is possible to obtain the Photoplethysmographic (PPG) signal [13] from the spectra of light reflected from (or transmitted through) body tissues. The Eulerian Video Magnification (EVM) method [17] has been proposed to emphasize these variations in a sequence of images captured as RGB (Red, Green, Blue) and transformed to YIQ (Y is the luminance component, while I and Q are the chrominance components), leading to the definition of the Videoplethysmographic (VPG) signal [18].

The aim of the work here presented is to propose a validation process of a contactless HR estimation approach, based on the processing of RGB images captured using Microsoft Kinect v2 [19,20,21,22,23,24] and enhanced by the EVM method, to amplify the changes in skin color due to blood flow. The choice of Kinect is motivated by the fact that it can provide additional information to RGB data, i.e., depth, infrared and skeleton frames. This capability can enable other applications, such as the monitoring of the physical activity and HR at the same time. Since the human face represents an area with uncovered skin, usually more exposed than other body areas, the monitored area is selected as a rectangle on the human face and neck; a face detection algorithm [25] is then used to select the correct Region of Interest (ROI). Due to the fact that the subject under test is in front of the acquisition device, face tracking is simpler and less prone to errors with respect to the tracking of other body parts, like the hands. Moreover, from the selection of the face area, it is possible to detect the neck region, where the presence of the carotid artery allows easier HR extraction. Then, a bandpass filtering process and an analysis based on the frequency spectrum [26] have been implemented to extract the HR value. The validation procedure has been carried out considering the HR value provided by a Holter, the gold-standard device for HR estimation. Finally, a commercial smartwatch has been used for comparison to the Kinect approach as a less expensive wearable solution.

The paper is organized as follows: Section 2 reviews approaches for unobtrusive HR estimation, whereas the proposed validation method is described in Section 3. Section 4 provides the implementation details of the method and presents the results in terms of HR estimation with the different approaches. Finally, Section 5 draws the conclusion.

## 2. Related Works

Several methods have been proposed in the literature to extract the HR in an unobtrusive way, and many of them are based on PPG [27,28] or VPG [29,30] signals. Medical devices based on PPG technology [31] (such as the pulse oximeter) are widely used in different healthcare applications, for example telemedicine and remote monitoring [32,33,34]. PPG has been also proposed for the analysis of Heart Rate Variability (HRV) [35,36] as an alternative to ECG, even if the method may be less accurate due to the difficulty in the detection of peaks in the measured signal.

The VPG signal [30] can be obtained through the analysis of the human face and neck; thus, a face detector may be used to select the ROI related to the human face and enable HR extraction in an unobtrusive way.

Viola and Jones [25] proposed a face detection algorithm from RGB images that has been used in many applications, based on an image representation named the integral image, which allows quickly calculating the face area. In order to select a limited number of critical visual features from a large series of potential characteristics, a strong classifier is constructed from a weighted combination of weak classifiers constituted by the integral image representations, each of which consists of a feature, a threshold and a polarity. A weighted combination of weak classifiers, followed by a threshold, provides a strong final classifier, based on the AdaBoost learning algorithm [37]. The “cascade” of classifiers allows one to filter out the background regions of the image and to focus the processing only on the candidate face regions. Verkruyss et al. [13] were the first ones, in 2008, to extract the PPG signal from a video (VPG), recording a face in stationary condition with a simple digital camera. The VPG signal was extracted from the values of individual pixels of the green channel from the entire face region and then filtered with a fourth order Butterworth filter. The HR was then obtained by applying the Fast Fourier Transform (FFT). In 2011, Poh et al. [14] introduced an automatic method for HR estimation exploiting face detection algorithms for the localization of the subject face and based on the study of the ROI, using a video recorded at 15 frames per second (fps) and with a 640×480 resolution. This method consists of averaging the pixel values of all three RGB channels in an ROI and the application of Independent Components Analysis (ICA) [38]. ICA is a statistical and computational technique for the separation of random variables (or signals) into non-Gaussian and mutually independent subcomponents.

Rahman et al. [39] compared three different signal processing methods for HR estimation: FFT, ICA and Principal Component Analysis (PCA) [40]. PCA is a statistical technique to extract a set of uncorrelated variables, called principal components, from a larger number of observations of possibly correlated variables. Thus, it can be used to reduce the dimensions or to enhance variations from a set of data. The algorithms were applied on RGB signals acquired from a webcam, considering 20 s of data at 30 fps. The system works in real time and includes signal acquisition, face tracking, ROI selection, signal detrending, filtering and normalization. Other applications, exploiting cameras integrated in portable devices such as smartphones, have been also proposed. Kwon et al. [6] developed a mobile application named FaceBEAT, for the remote HR measurement from videos of the human face.

Many systems have been proposed to assess the performance of HR estimation algorithms based on video data. Monkaresi et al. [7] proposed a method for HR extraction based on ICA using video data. The population considered for the test is constituted of 10 subjects, eight males and two females, instrumented with Electrocardiogram (ECG), respiration and Galvanic Skin Response (GSR) sensors to validate the method. The approach proposed by Poh et al. [14] was evaluated under two new conditions: naturalistic human computer interaction and exercising scenarios, with better results thanks to the adoption of a machine learning approach. In the former one, the authors had to cope with the variability of face illumination in the displayed content, which can cause domination of red or blue components leading to almost undetectable variation in the green channel. A method based on data from the Kinect device [41] was introduced in conjunction with a processing algorithm to calculate Respiratory Rate (RR) and HR and a comparison to reference methods: spirometry and ECG, respectively. Results from the tests performed on 10 healthy subjects show that the Kinect is a valid contactless device for the monitoring of HR and RR at home, without the presence of experts or clinicians. Iozzia et al. [42] proposed a fully-automated method based on the chrominance model working on RGB images of the human face. Three different ROIs (forehead, nose, cheeks) were selected to detect and evaluate the accuracy of beat detection and InterBeatIntervals (IBI) measurements. The experimental recordings were conducted on subjects under two different conditions: resting and standing. The validation with a three-electrode ECG let them conclude that it is not possible to consider a specific ROI that gives better results, but the optimal selection appears to be inter-subject and posture dependent. Procházka et al. [11] recorded image and infrared data of facial features for HR, and depth data of the thorax region for RR, using Kinect. In particular, three different ROIs in the mouth area have been considered. The results in terms of HR estimation have been compared with a wearable sensor (Garmin) demonstrating that the Kinect device can be used to detect sleep abnormalities. Data from video sequences can be also processed using Short-Time Fourier Transform (STFT) [43] to reveal the subject’s HR. Thanks to STFT, it is possible to provide temporal and frequency information that is more accurately localized, especially for the rapidly changing HR pattern during the exercise routine.

The EVM method [17] has been introduced to amplify the small variations in skin color, which are not normally visible to the human eye, through spatial and temporal filtering techniques. EVM differs from ICA because it employs localized spatial pooling and temporal filtering to extract the signal of the cardiac pulse [44]. EVM extracts the VPG signal from the YIQ color space rather than RGB. Smilkstein et al. [45] proposed the use of EVM to estimate HR from a Kinect mounted on the car’s dashboard, in order to monitor the driver. The work we present is similar to [45], since it is based on the same data acquisition technology (Kinect) and processing technique (EVM). However, they selected a narrow interval for admissible HR values, from 50–100 bpm. In our work, on the contrary, the interval of acceptable values is larger, thanks to the introduction of the a priori knowledge about the normal HR of the subjects. A recent study [46] demonstrated that it is possible to extract the HR using an adaptive approach that simultaneously finds the best ROI for HR estimation. This method, called self-adaptive matrix completion, with a principled data-driven approach to automatically detect the face parts, is useful for HR measurement. It is based on the estimation of the time-varying mask of useful observations, selecting at each frame the relevant face regions from the chrominance features themselves to compensate for illumination variations.

## 3. RGB Processing for HR Extraction

**Figure 1:**
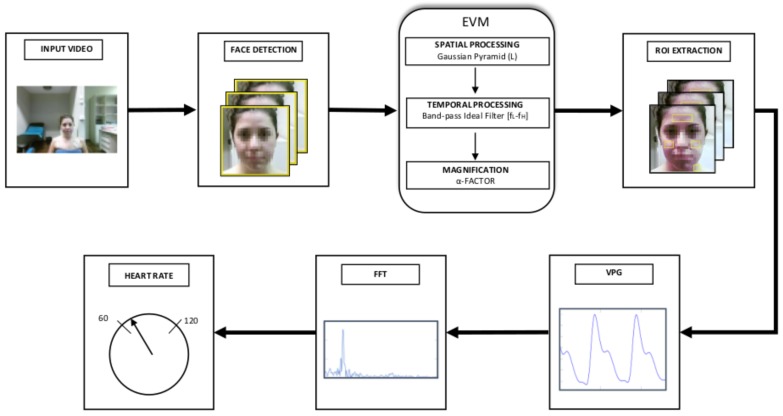
The main scheme of the proposed system for the computation of HR from the RGB video.

The proposed method for the estimation of HR takes as input RGB frames of a human face. The area related to the human face is detected and processed by the EVM algorithm [17], which enhances the small changes in luminance due to blood flow. Then, limited areas of the face and neck are selected through an ROI extraction step. Finally, the main frequency component of the VPG signal within the ROI is computed by FFT, and the HR value is the output of the system. The main scheme of the method is shown in Figure 1, where the different steps can be identified.

The method requires that the human subject is in front of the acquisition device, and the face has to be included in the input frames. The first processing step is about the identification of the area related to the face and the neck, since it is necessary to reduce the data that have to be processed by the EVM method. This first task is implemented by applying the well-known Viola–Jones algorithm [25] to locate the face area. The face and neck are then considered as objects to be tracked and their positions are extracted in the subsequent frames by the Kanade–Lucas–Tomasi (KLT) feature-tracking algorithm [47,48] based on features extracted through a minimum eigenvalues algorithm [49].

**Figure 2:**
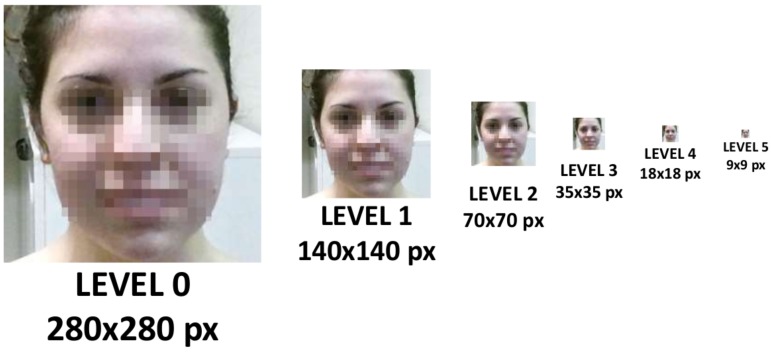
Spatial decomposition through the Gaussian pyramid of five levels with specific details on the pixel resolution. Since the dimensions of the square detected face area are not integer exponents of two, the size is rounded to the nearest integer.

The following EVM processing encompasses three steps, i.e., spatial and temporal processing, together with a signal magnification. Spatial processing consists of the decomposition of the frames with a Gaussian pyramid, considering a number of predefined levels *L*. The Gaussian pyramid is created from each image featuring the human face, including the neck, by blurring and downsampling it into various levels. In fact, the spatial pyramid is a set of layers in which the image is downsampled so that the higher layer has a smaller size, as can be noticed from Figure 2.

Following spatial decomposition, a temporal processing is implemented on each spatial band to extract the frequency bands of interest. In fact, the implementation of the EVM method for different applications may require considering different frequency bands. For noise reduction, narrow temporal bandpass filters are preferred whilst motion amplification needs a broader filter [17]. Ideal or Infinite Impulse Response (IIR) bandpass filters can be applied both for motion and color amplification, extracting the interval between the cutoff frequencies $f_{L}$ and $f_{H}$.

The last step is necessary to amplify the signal through the magnification factor (α), which has to be carefully chosen to avoid the presence of artifacts in the video. This is a multiplying factor used to enhance the visual identification of the changes in color due to blood flow. Since the processing is implemented in the YIQ color space, this phenomenon can be attenuated by reducing the α factor that multiplies the chrominance components I and Q.

**Figure 3:**
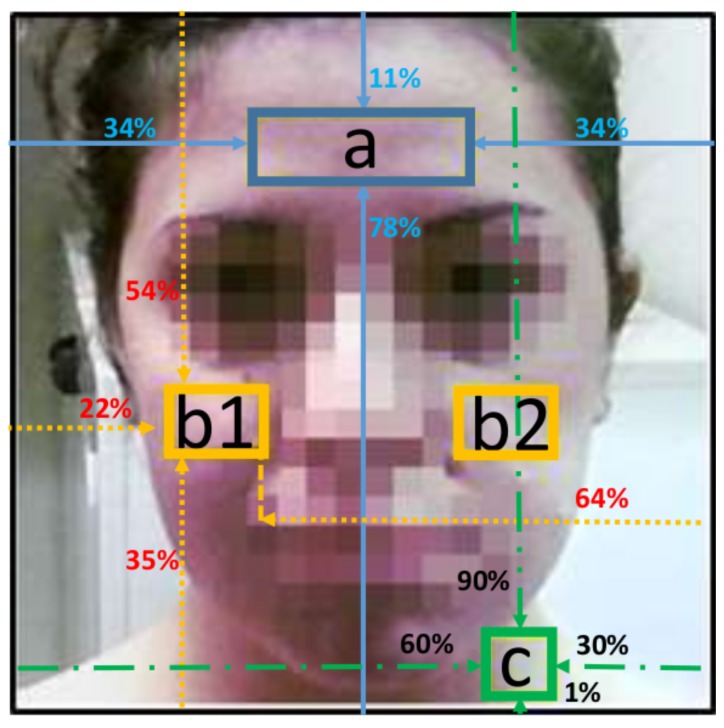
Selected regions of interest: (**a**) Forehead (F); (**b1**,**b2**) Cheeks (C); (**c**) Neck (N). Some details are blurred to preserve the subject’s privacy. The ROIs are selected considering the percentage of the detected face area shown in the figure.

Once signal magnification of the face area through the EVM method is completed, the magnified set of frames is processed to select some regions of the face, from which the VPG signal is extracted. The ROIs are related to the areas of Forehead (F), Cheeks (C) and Neck (N), as can be seen in Figure 3, and they have been chosen considering that in these areas, the skin is usually exposed. The pixels inside the ROIs are converted from RGB color space into the YIQ color space. The VPG signal is constituted by the average values of the Y-component over all of the pixels of the selected ROIs, taken individually or jointly, for all of the frames (i.e., 1200 frames for a sequence of 40 s captured at 30 fps) processed by the EVM algorithm. Using the mean luminance values of the ROI in each frame is justified by the meaning of the luminance as the intensity level of the three chrominance components. This average calculation has advantages in increasing the signal-to-noise ratio to a useful level.

**Figure 4:**
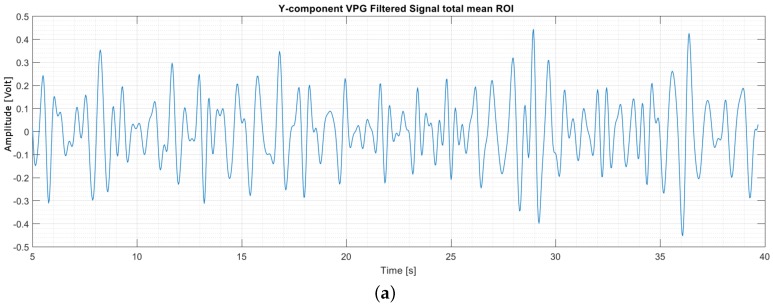

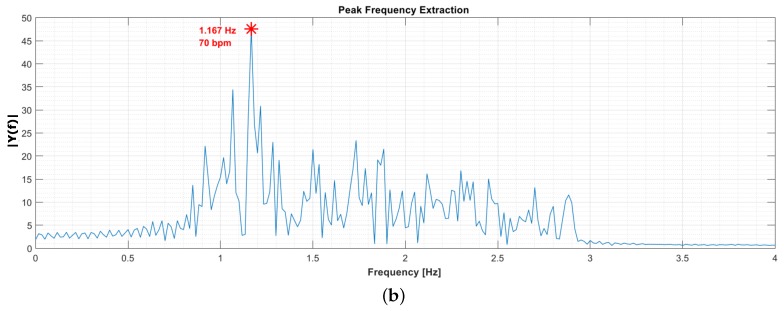
VPG signal (Y-component) obtained by averaging the signals of all of the ROIs (F, C, N or, rather, ROI T (Total)) over one test execution. The signal is represented in the time domain (**a**) and in the frequency domain (**b**), after the bandpass filtering process.

After the extraction of the VPG signal in the time domain, as shown in Figure 4a, and its transformation in the frequency domain, through the application of FFT, take place, the idea is to extract the frequency component of the VPG signal that is dominant in a specific band, which is related to the blood circulation and thus to the HR. A Butterworth bandpass filter is applied to select the band of interest, and a spectral analysis is performed to extract the main frequency component, as can be noticed from Figure 4b. Finally, since the HR is expressed in beats per minute (bpm), the final operation is the straightforward conversion of the dominant frequency component into the bpm scale.

## 4. Validation

**Figure 5:**
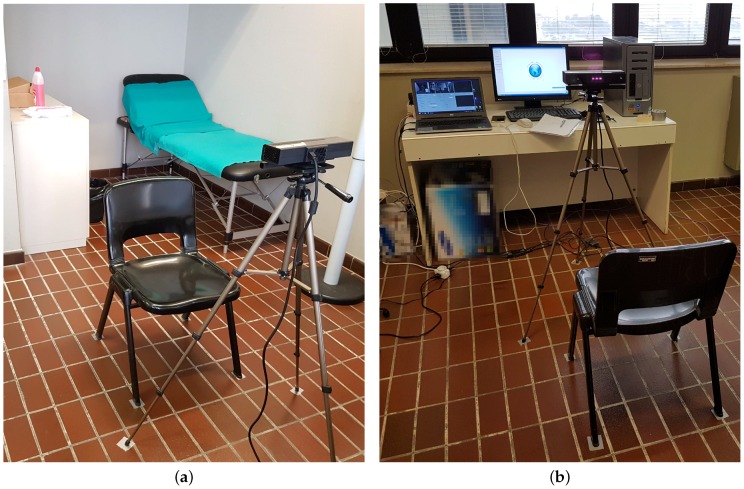
Laboratory setup at Università Politecnica delle Marche, where the tests have been conducted. Two different environmental conditions are shown: high (**a**) and low (**b**) light.

The proposed method based on EVM is validated through the comparison of the HR estimated value with the result provided by a Holter device. Then, the estimated HR value provided by the RGB-based system is compared also to a commercial smartwatch. The experiments have been conducted in a laboratory environment at Università Politecnica delle Marche, with the setup shown in Figure 5.

### 4.1. Data Acquisition and Processing

The data acquisition setup is constituted by different sensors, and the Microsoft Kinect v2 is the one that provides the RGB frames needed by the EVM method. The raw RGB frames captured by the sensor are stored as bitmap images, with a resolution of 1920×1080 and a frame rate of 30 fps, through the capture software named Complete Viewer v2.0 (http://www.tlc.dii.univpm.it/blog/databases4kinect), and after the application of the face detection process, they are used as input to the EVM.

The gold-standard instrument is represented by the Global Instrumentation’s M12A (http://www.globalinstrumentation.com) Holter, which extracts the ECG using the electrodes placed on the human body [50]. Among the 12 leads available from the Holter, only the aVR one is considered for HR extraction. The sampling frequency of the device is 1 kHz, and a frequency band between 0.05 Hz and 100 Hz is selected with a sixth order Butterworth filter. The algorithm proposed by Pan and Tompkins [51] is used to extract the R-R peaks and estimate the HR mean value. The dedicated laboratory of the university where tests have been conducted is far from significant electromagnetic sources, so we can consider the certified Holter as a reliable device. A single test has a duration of 40 s, and we can assume that in this time interval, the HR estimation provided by the Holter is accurate. In fact, at rest and on a 40-s interval, the HRV is limited.

The second device used to compare the HR extracted values is the commercial Moto 360 smartwatch by Motorola. The smartwatch does not provide raw data about heart activity, but it exploits the PPG technique to compute the HR value. We used an app for Android smartwatches, called LG Pulse, that allows one to synchronize the acquisition time with the other devices. Indeed, from the moment at which the app is started, we synchronize the others’ acquisitions, by introducing a synchronization burst to enable the time alignment of the VPG and ECG signals.

A number of 20 young subjects participated to the validation tests (age: 22.50 ± 1.57; height: 173 cm ± 10; weight: 62.80 kg ± 9.52; BMI: 20.96 ± 1.88), and each subject repeated the test five times, generating a total number of 100 sequences. For practical reasons, subjects were facing the Kinect while sitting on a chair at a distance of about one meter considering that the horizontal field of view of the device is 70${}^{{}^{\circ}}$ and the vertical field of view is 60${}^{{}^{\circ}}$. The acquisition time has been set to 40 s. Experiments have been carried out with different light conditions, and the light intensity has been measured by an Android application (Light meter) running on a Sony Z2 smartphone. The smartphone was placed next to the human face before starting the data acquisition to estimate the environmental light. The aim of the test was to verify the variability of the HR extraction under different lighting conditions, so relative lighting measurement was considered adequate. Tests were performed indoors with artificial light, but the natural light coming from the windows changed the intensity of the ambient light at different times of the day, as Figure 5a,b show. Therefore, the subjects have been exposed to variable light conditions.

Regarding our proposed system, some parameters have to be set. The face detection algorithm working on image frames can be tuned to avoid the identification of small objects associated with faces. To this aim, the minimum size of the face has been set as a square with a dimension of 280×280 pixels, ensuring the inclusion of the subject’s face and neck. The ROIs of the forehead, cheeks and neck, shown in Figure 3, have been also set respectively to 90×30, 40×30 and 28×26 pixels. ROI sizes were empirically selected considering all of the test sequences, thus involving a large number of subjects, and setting the percentages of box identification shown in Figure 3 with respect to the area of the face detector.

The EVM parameters, required by the open source code provided by MIT, http://people.csail.mit.edu/mrub/evm/, are chosen as follows. The Gaussian pyramid adopted for spatial decomposition is characterized by six levels and a binomial filter for blurring. Temporal processing is implemented with an ideal bandpass filter that can select three different intervals depending on the implemented approach. In fact, the system based on the Kinect has been considered with two different approaches, which can be defined as supervised or unsupervised, depending on the use of the a priori knowledge about the subject lifestyle. This knowledge, included in the supervised approach, allows one to select an interval of admissible HR values for people that are physically active, which is different from the one used for inactive adults. In particular, in the supervised approach, the interval of admissible values has been set to (30–174) bpm for athletes and to (66–174) bpm for non-athletes, corresponding to the definition of two band-pass filters with lower cutoff frequencies of 0.5 Hz and 1.1 Hz, respectively. The definition of a larger interval of frequencies than the range of bpm values shown in Table 1 is motivated by the fact that the cutoff frequencies of the filter should be chosen to allow the detection of the main frequency component with a guard interval. The higher HR interval, used for non-athletes, can be used also for subjects with diseases, or features generating high heartbeats (such as low blood pressure or tachycardia). On the other hand, the term unsupervised is associated with the second approach that does not rely on the knowledge about the subjects’ lifestyle. In this case, a unique interval of (50–174) bpm has been considered for admissible values for HR, corresponding to a bandpass filter set to (0.83–2.9) Hz, for athletes and non-athletes. The participants are classified as athletes or non-athletes according to their own statement on the informed consent form delivered prior to testing and also by checking their HR at rest with Holter measurement. To synchronize the heart rate estimation with the Kinect, Holter and smartwatch, a synchronization burst is introduced at the beginning and at the end of the acquisition time, which produces in the Y-component of the VPG signals a disturbing noise. Even in the presence of this noise, in the supervised approach, the filtering, at the different chosen cut-off frequencies, gives rise to a sufficient signal to noise ratio in order to achieve an adequate accuracy in the HR extraction. On the contrary, in the unsupervised approach, where a trade-off bandwidth filtering is considered, it is suggested to remove the first and the last samples to have a suitable signal to noise ratio in the overall range of the admitted values of HR. This requires that 10 s of data to be discarded from the entire set of frames, 7 s at the beginning and 3 s at the end; it has been verified during the laboratory tests that the Holter measurements of the HR are not affected by this reduction of the observation time.

A third order Butterworth filter, with the same cutoff frequencies of the ideal filter, is applied to extract the band of interest from the VPG signal, extracted from the contributions of the selected ROIs. The magnification factor α is set to 100 to have a good compromise between signal amplification and the presence of artifacts.

### 4.2. Experimental Results

The systems for HR estimation considered in this work, i.e., Holter, Kinect v2 and a smartwatch, were compared considering the Holter as a reference. The error term is defined as the absolute difference in the estimation of HR, and it is expressed as percentage, as reported in Table 2. Regarding the approach based on EVM working on RGB data from Kinect, Table 2 shows results obtained from different ROIs, and T (Total) is the average of all of them. The errors included in Table 2 are the mean errors over all 100 tests, considering that the average measured HR is 77 bpm. All signals are processed in the MATLAB environment.

The results show that, compared to the Holter data, the error provided by the smartwatch-based HR estimation is 7.18%.

Regarding the performance of our method, the consideration of a VPG built by averaging values obtained from different ROIs allowed reducing the noise and increasing the SNR. Both supervised and unsupervised approaches have been considered to validate the system based on VPG. The method has been implemented considering the parameters described in the previous section.

The best result, represented by an error of 2%, has been obtained with the supervised approach on the ROI labeled as T, which requires considering the contribution from all of the ROIs: forehead, cheeks and neck. The contribution from all of the ROIs is calculated by averaging the luminance in the total area obtained adding each single ROI. The unsupervised approach was able to achieve a minimum error of 3.4% with the same ROI. If the ROI contributions are considered separately, the area related to the forehead is the one featuring the higher error, for both approaches (4.13% for supervised and 7.78% for unsupervised), as can be seen from Table 2. In fact, the forehead area is the most exposed to the light, which may introduce noise in the VPG signal generated by EVM. Cheek and neck ROIs feature a lower error in HR estimation, which is around 3% for the supervised method and 6.5% for the unsupervised one. This is because the neck area contains the carotid artery and the presence of blood vessels in the area related to cheeks. The ROI named N + F considers the joint contribution of neck and forehead, and it is able to improve the results of the separated regions, bringing an error of 2.13% for supervised and 5.55% for unsupervised.

Table 3 provides a more detailed overview of the results obtained with RGB data from Kinect, since it includes the average results for each subject. Subjects are grouped considering their lifestyle (according to their own judgment) by assigning the label A to athletes and NA to all of the other subjects, and the results for all of the selected ROIs and two different approaches are shown. The comparison of the results included in Table 3 highlights an error that is lower for the supervised approach in both the subject groups: Athletes (A) and Non-Athletes (NA). Furthermore, the results considering different ROIs confirm the conclusion derived from Table 2, since the minimum error is represented by ROI T, and the maximum one is given by ROI F. For example, the unsupervised approach using the forehead ROI is characterized by the maximum error in both populations, which is 5.83% for A and 8.27% for NA. The use of the supervised approach allows reducing the error, giving a percentage of 4.95% for A and 3.89% for NA. Moreover, this approach allows using the entire sequence of frames, which has a length of 40 s, for the extraction of the VPG signal. In fact, many noise contributions are filtered by using $f_{L}=1.1$ Hz in most of the cases (NA subjects) and $f_{L}=0.5$ Hz only when a larger frequency band is required (A subjects). In the unsupervised approach, where the frequency band is fixed and $f_{L}=0.83$ Hz is considered for each subject, there is the need to remove some noise contributions, which are localized at the beginning and at the end of the VPG signals. Thus, a subset of frames is considered to extract the VPG signal by removing the first 7 s and the last 3 s from the entire sequence, leading to the consideration of a time sequence of 30 s in the unsupervised approach. Finally, it can be stated that, even considering the unsupervised approach with the ROI related to the forehead, which is the simplest test configuration, the average error on 100 sequences acquired in different environmental conditions is lower than 8%.

**Figure 6:**
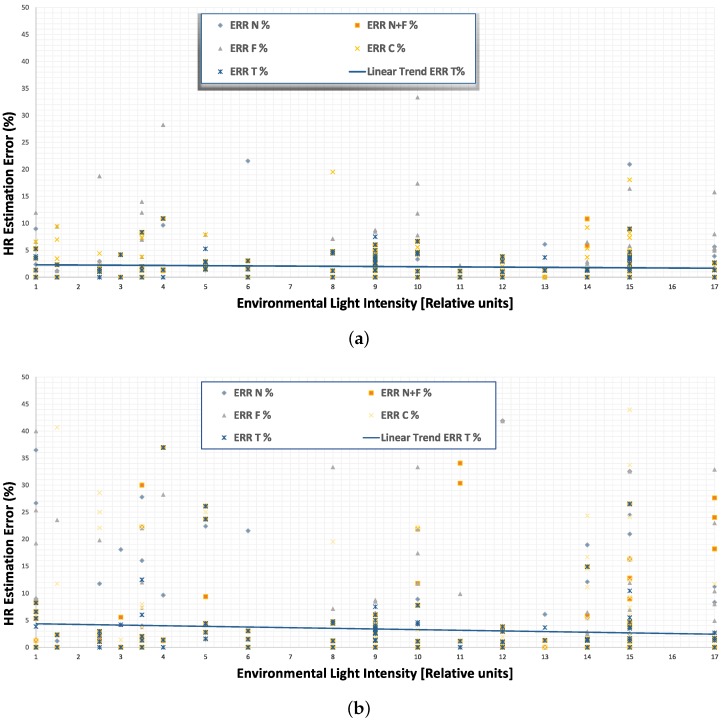
Average error (%) in HR estimation for the system based on RGB data with different light conditions on different ROIs; (**a**) supervised approach; (**b**) unsupervised approach.

The relation between HR estimation error and the environmental light conditions has been also investigated for the system based on RGB data from Kinect, and the results are summarized in Figure 6a,b, respectively, for the supervised and unsupervised approaches.

The scatter graphs include the results for each test in addition to the linear trend lines, revealing the dependence between relative intensity of light and error in HR estimation. The figures show these trends only for the total ROI (T), even if the same conclusion can be drawn for the other ROIs. The intensity of environmental light (natural and artificial) influences the HR estimation. Both approaches (supervised and unsupervised) benefit from the increasing of environmental light, even if the unsupervised approach shows a higher gradient of error. Considering the supervised approach, the higher the light intensity, the better the HR estimation, even if a good HR estimation can be obtained also with low environmental light. This behavior suggested to us that the Kinect device is also suitable for different applications in low-light conditions.

The unsupervised approach can lead to the generation of some artifacts in the image (especially in the forehead area) that are translated into a noisy VPG signal. Here, the gradient of error given by total ROI in terms of light intensity is larger, which means that having an environment with enough light is more important if the unsupervised approach has to be chosen. In conclusion, the supervised approach is able to compensate the variations due to the environment, leading to a low error in HR estimation in low light conditions.

**Figure 7:**
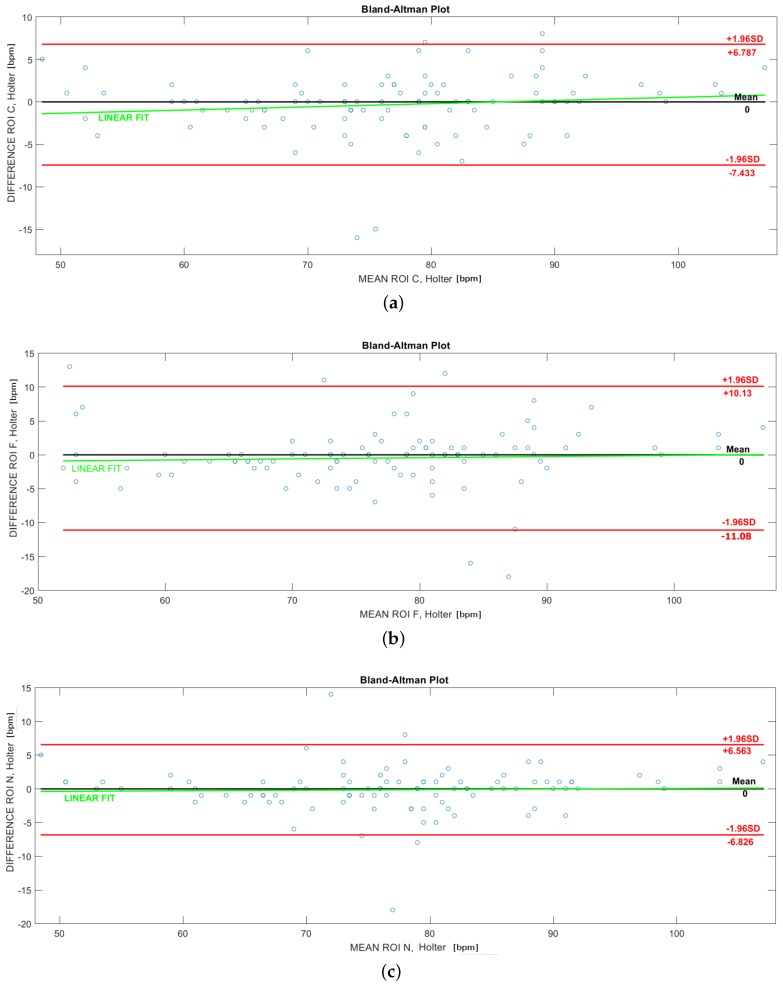
Bland–Altman plot, correlation between HR values extracted by the Holter and through the proposed method in: (**a**) ROI C; (**b**) ROI F; (**c**) ROI N.

**Figure 8:**
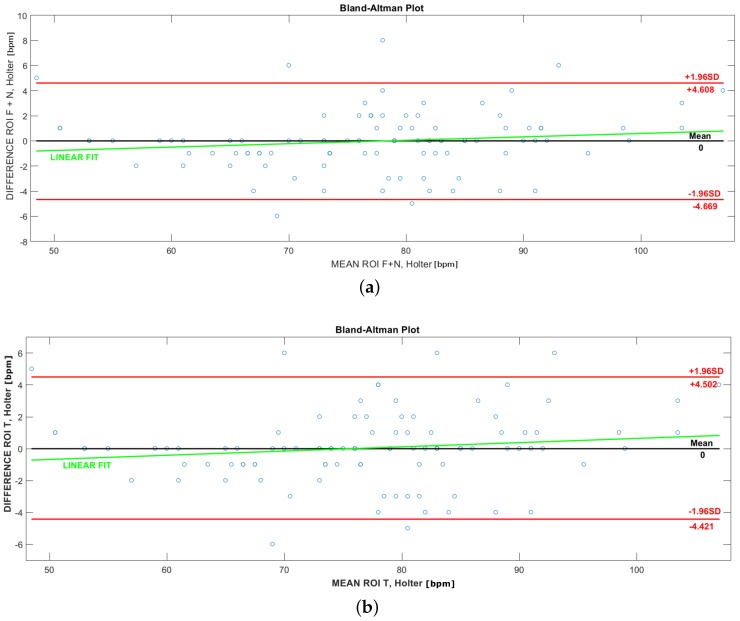
Best correlation values with the Bland–Altman plot, between HR values extracted by the Holter and through the proposed method in (**a**) ROI F + N; (**b**) ROI T.

To evaluate the correlation between the two measurement techniques (wearable and contactless) compared in this work, the Bland–Altman bias plots [52], shown in Figure 7 and Figure 8, are provided. Each estimated HR value is compared to the value obtained with the Holter monitor (not averaged values). The statistical values have been calculated as the mean and the Standard Deviation (SD) of the differences between Holter and VPG measurements. The 95% (from −1.96 SD to +1.96 SD) limits of agreement for the difference values and the corresponding mean value are shown in both Figure 7 and Figure 8a,b (red line). Table 4 shows the Root Mean Square Error (RMSE) and $r^{2}$ (R-squared) values between the Holter and the extracted HR values in ROIs. R-squared denotes how well the regression line approximates the real data points. An R-squared of one indicates that the regression line perfectly fits the data. Figure 7a–c show the plots of the difference between the Holter and the extracted HR values in ROI C, ROI F and ROI N, respectively. Figure 8a,b are the best plots representative of the minimum error between the Holter and the HR extracted from FFT in the areas respectively of the: ROI N + F and ROI T. As the plots confirm, the best correlation, shown in Figure 8b, is between the Holter and ROI T.

**Figure 9:**
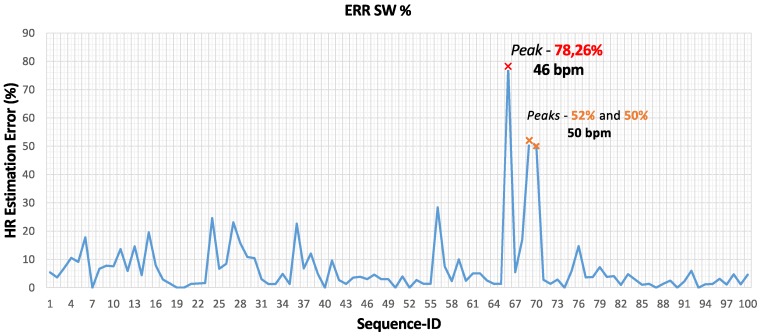
Average error (%) in HR estimation revealed by the Smartwatch (SW) with respect to Holter measurements.

Figure 9 shows the error values provided by the smartwatch and the gold-standard. The smartwatch features a large error at Sequence-ID 66, which is associated with an athlete with an HR of 46 bpm. It can be observed that the HR estimation error is greater than 30% only in three sequences featuring an HR value lower than 50 bmp.

## 5. Conclusions and Future Works

The signals generated by a Microsoft Kinect may be exploited in many different applications, like in contactless HR detection. This would make possible, for example, the indoor monitoring of subjects in need of a holistic, constant and economic control of their life conditions (food intake monitoring, sleep monitoring, HR monitoring). To this aim, we provided a performance evaluation of a contactless and Kinect-based non-biomedical system to monitor the HR in non-clinical environments. The validation of the EVM method based on RGB data extracted from Kinect demonstrated that it is possible to estimate the HR of a subject in an unobtrusive way, without any contact, with an acceptable error. This error can be further reduced if some knowledge about the lifestyle of the subject is introduced in the system. In fact, physically-active subjects feature a normal HR, which is lower than inactive people. This information allows one to properly choose the parameters used by the HR estimation algorithm, thus increasing the accuracy of the system.

This project will be further developed to allow HR estimation while the subject is performing body movements, for example during rehabilitation exercises.

An integrated system for the monitoring of body motion and biomedical parameters should be implemented in the future, for a global analysis of the subject’s health conditions.

## Figures and Tables

**Table 1:** Typical range of normal heartbeat for different age groups [1].

Age	Heart Rate (bpm)
Infants	100–180
Children	80–100
Teens	70–120
Adults	60–80
Athletes	40–60

**Table 2:** Comparison among different methods (based on Kinect and wearable devices) for HR estimation, in terms of average error (%). The average HR over the 100 signals measured with the Holter is 77 bpm. N + F is the mean value of the sum of the N and F ROIs; T is the mean of the sum of all the ROI, identified as Total ROI; SW stands for Smartwatch.

Approach	ROIs from Kinect RGB Data					Wearable
	F	C	N	N + F	T	SW
Supervised	4.13%	3.10%	2.70%	2.13%	2.00%	7.18%
Unsupervised	7.78%	6.55%	6.69%	5.55%	3.40%	

**Table 3:** Average error (%) in HR estimation for each subject with RGB data from the Kinect and Holter average values, with the supervised and unsupervised approach. The subjects are grouped considering their lifestyle as Athletes (A) and Noon-Athletes (NA).

Subject ID	Supervised					Unsupervised					Holter (bpm)
	F	C	N	N + F	T	F	C	N	N + F	T	
05-A	2.15	1.68	0.95	1.48	0.61	2.71	2.23	1.17	2.04	1.17	65.40
08-A	3.68	2.36	1.71	1.33	1.33	3.61	2.29	1.97	1.59	1.59	58.60
10-A	13.06	6.38	2.97	2.97	2.97	16.11	11.97	9.59	8.05	8.56	50.80
20-A	0.90	1.20	5.81	1.20	1.20	0.90	1.20	5.51	1.20	1.20	66.20
avg-A	4.95	2.91	2.86	1.75	1.53	5.83	4.42	4.56	3.22	3.13	60.25
01-NA	1.92	2.20	4.12	2.44	2.20	5.89	5.89	10.68	6.19	3.46	76.40
02-NA	14.29	1.97	1.75	2.42	2.42	12.18	4.70	2.26	2.04	2.04	91
03-NA	1.59	1.15	1.37	1.37	1.15	4.57	0.87	1.31	15.47	1.09	88
04-NA	3.32	3.61	2.01	2.27	2.79	15.52	7.40	16.23	17.08	12.07	69.80
06-NA	2.06	4.97	5.13	1.64	2.08	6.07	20.69	13.69	5.85	6.73	85.20
07-NA	1.85	1.56	2.28	0.82	1.55	3.49	6.28	8.34	3.37	2.24	90.60
09-NA	4.68	3.06	3.86	3.08	2.79	15.24	3.56	14.22	4.03	4.03	77.40
11-NA	6.88	1.85	2.71	1.85	0.79	17.25	3.80	7.64	20.23	2.26	74.60
12-NA	5.69	6.38	4.22	4.22	4.22	12.90	8.80	11.67	6.72	4.52	79
13-NA	0.76	2.09	2.41	2.41	0.25	1.00	4.61	1.01	0.75	0.49	77.60
14-NA	6.08	4.60	0.87	0.89	0.89	10.18	26.07	4.88	3.62	3.62	89
15-NA	1.71	1.72	1.42	1.42	1.13	1.58	1.57	3.84	1.86	1.29	70.40
16-NA	4.51	3.76	3.50	3.76	3.76	4.59	7.66	5.60	3.84	3.84	79.80
18-NA	2.58	3.58	2.56	2.83	3.58	2.34	2.84	2.32	2.09	2.84	101.40
17-NA	1.75	1.55	1.75	1.75	1.75	10.42	2.06	9.62	2.25	2.25	77
19-NA	2.56	5.98	2.08	2.08	2.08	9.07	6.72	2.82	2.82	2.82	83.80
avg-NA	3.89	3.13	2.63	2.20	2.09	8.27	7.10	7.26	6.14	3.47	81.94

**Table 4:** RMSE and R-squared values.

ROI	RMSE (bpm)	R-Squared
ROI F	5.2	0.81
ROI C	3.5	0.91
ROI N	3.37	0.92
ROI N + F	2.3	0.96
ROI T	2.2	0.97

## Abbreviations

The following abbreviations are used in this manuscript:

bpm	Beats per minute
ECG	Electrocardiogram
EVM	Eulerian Video Magnification
FFT	Fast Fourier Transform
fps	Frames per second
HR	Heart Rate
HRV	Heart Rate Variability
IBI	InterBeat Intervals
ICA	Independent Components Analysis
PCA	Principal Components Analysis
PPG	Photoplethysmography
VPG	Videoplethysmography
ROI	Region Of Interest
RR	Respiratory Rate
SNR	Signal to Noise Ratio
RGB-D	Red, Green, Blue-Depth
YIQ	Y Luminance, I and Q Chrominance
IIR	Infinite Impulse Response
RMSE	Root Mean Square Error
SD	Standard Deviation
